# Patients’ perceptions of orthodontic treatment experiences during COVID-19: a cross-sectional study

**DOI:** 10.1186/s40510-021-00363-7

**Published:** 2021-06-08

**Authors:** Sarah Abu Arqub, Rebecca Voldman, Ahmad Ahmida, Chia-Ling Kuo, Lucas Da Cunha Godoy, Yousef Nasrawi, Susan N. Al-Khateeb, Flavio Uribe

**Affiliations:** 1 Division of Orthodontics, University of Connecticut Health, 263 Farmington Ave, Farmington, CT 06032 USA; 2 Connecticut Convergence Institute for Translation in Regenerative Engineering, University of Connecticut Health, Farmington, CT USA; 3 Sunna Orthodontic Center, Amman, Jordan; 4 Jordan University of Science and Technology, Irbid, Jordan

**Keywords:** COVID 19, Orthodontic Clinic, Patient psychology, Strict protocol

## Abstract

**Background:**

COVID-19 has impacted the care of patients undergoing orthodontic treatment. We aimed to provide an overall view of patients’ perspectives, concerns, and expectations towards their treatment throughout the clinic lockdown during the pandemic; and to assess patients’ levels of mental distress and its association with their confidence in resuming care.

**Methods:**

An anonymous, validated, in-person paper questionnaire was distributed to adult orthodontic patients’ post-lockdown at an academic institution. The survey focused on the clinical aspects and patients’ perspectives regarding orthodontic treatment during the pandemic. The Kessler Mental Distress Scale (K10) was used to evaluate their psychological status. Survey responses were descriptively summarized and confidence in resuming care was compared between normal patients and patients with mental distress using Mann-Whitney tests.

**Results:**

One hundred fifty-four patients were surveyed from July to October 2020. Mean age of the participants was 29.30 (SD = 12.01) years and 62% were females. Emergencies during the closure (21%, 32/154) involved primarily irritation with protruding wires. Patients were neutral regarding tele-dentistry and preferred their current fixed appliances over clear aligners. Upon resuming care, 80.51% were extremely pleased with the restrictive protocols and with high level of confidence in resuming treatment. The average level of anxiety was low, and a modest association was found between mental distress and reduced confidence in resuming treatment.

**Conclusions:**

Few numbers of minor emergencies occurred during the clinic closure. Despite the rising interest in tele-dentistry, patients were neutral on considering this option to monitor treatment and were content with fixed appliances. Patients had high confidence levels to resume their care based on the protocols established upon reopening. The association of mental distress and confidence in resuming care is suggestive and needs further investigation.

## Background

The COVID-19 pandemic disrupted every aspect of our society. New circumstances related to specific requirements for contamination and cross infection control were enforced in each profession [1, 2]. The Occupational Safety and Health Administration (OSHA) classified dentistry as a “very high risk” profession [3]. This was due to the nature of the virus and its infectious route which could spread through airborne droplets in the form of aerosols. This disclosed certain potential hazards underlying conventional and standard oral health care procedures [1]; thus, guidelines and safety protocols for dental practices in the COVID-19 era were established [4]. Despite the fact that documented cases of cross infection in US orthodontic offices are extremely rare [5], orthodontists are still health care providers and therefore these safety recommendations require to be followed as well.

Unlike other elective dental procedures, orthodontic treatment is an ongoing process that relies mainly on consistent evaluation and adjustment of active appliances throughout treatment [6]. Many patients were undergoing active treatment when their care was suddenly suspended because of the pandemic.

Evaluation of anxiety and psychological distress is necessary to monitor and determine the trends in mental health for both individuals and populations over time. The Kessler Psychological Distress Scale (K10) was developed in 1992, in the annual US National Health Interview Survey to measure the non-specific aspects of psychological distress (see http:// www.hcp.med.harvard.edu/ncs/k6_scales.php for more detail) [7, 8]. The quantity of research that has eventually utilized the (K10) scale is immense and includes health evaluation studies and intervention outcome measurement in both the medical and dental fields. For example, the (K10) scale has been used in samples of advanced cancer [9], individuals with HIV [10], epilepsy [11], and more recently in studies evaluating the psychological impact of COVID 19 in various populations and fields including orthodontics [12–18]. Xiong et al [19] recently demonstrated using the Kessler Psychological Distress Scale (K10), that over one-third of the orthodontic patients experienced mental distress during the early stage of the pandemic in China. Many factors played a role in this distress, including the type of appliance, intervals between visits, and channels of communication with orthodontists. A relationship has been established between long-term isolation and the development of anxiety and depression [20]. Understanding the psychological aspects that are relevant to clinical orthodontics and how patients perceived their care during lockdown and as they resume treatment remains elusive. Additionally, some orthodontic treatment involves aerosol-generating procedures [21]. The risk of disease transmission in orthodontics and the role of orthodontic patients as “asymptomatic carriers” is still unknown and may pose a potential elevated threat [22].

An in-depth elucidation of orthodontic patients’ perceptions and concerns regarding treatment during these unprecedented times is important. It would be helpful to understand what issues were faced during the lockdown, how they communicated with their providers in case of emergency care, and their perception of their progress and future continuation of treatment. Furthermore, clinic setup, safety plans, and personal protective equipment (PPE) protocols should be examined for insights into the patients’ perspective. Being that patients’ safety and satisfaction is always a priority, their attitudes towards clinical protocols and safety plans implemented give the providers an insight into the effectiveness and quality of the clinical care provided.

This study is even more imperative now since once again numerous countries are still experiencing an alarming post-lockdown resurgence of the pandemic and a new viral strain, triggering fears for a devastating second pandemic wave [23]. The aim of this study was to assess orthodontic patients’ perceptions, concerns, and expectations towards their treatment during this pandemic, to assess patient’s levels of mental distress, and to test for associations between their psychological mental distress and confidence in resuming orthodontic care. Our null hypothesis is that the pandemic has no effect on the psychology of orthodontic patients and on the attitude of orthodontic patients towards continuing treatment.

## Materials and methods

The procedures and protocol for this cross-sectional descriptive study were approved by the institutional review board (21X-011-1). An in-depth review of the literature of some of the critical aspects related to the patients’ concerns that are relevant to orthodontic practices during the pandemic was conducted to develop the survey instrument in this study [17, 18, 21, 24–26]. A preliminary survey that included 7 sections and 42 questions was evaluated by ten consultant orthodontists for content validity, five were from the private practice sector with more than 5 years of clinical experience, and five were associate professors in an academic institution. Each question was evaluated on a 3-point scale (not necessary, useful non-essential, and essential). Content validity ratio (CVR) was then calculated for each item according to “Lawshe’s Method” [27]. Two items were not significant at the critical level; thus, were eliminated.

The final survey instrument was in paper format and in person. It consisted of 40 questions divided into 7 sections. A cover memo explaining the aims and objectives of the survey and highlighting that taking the survey is voluntary was attached to the survey.

Section 1 assessed the demographics; age, sex, and general information about the treatment duration and length of time the patient was not seen by an orthodontist during the pandemic lockdown. Section 2 included questions related to the psychological aspects relevant to clinical orthodontics; patients’ expectations on the standard of care delivered, time needed to complete their treatment, level of commitment to oral hygiene practices and post-operative appliance instructions, pain or discomfort felt, and their presumption on the delay of treatment caused by the pandemic. Section 3 focused on how orthodontic emergencies were managed; if any was encountered; nature of the emergency, degree of the severity, and the choice of handling emergencies. Section 4 focused on the patients’ preferences regarding the urgency on following up treatment during the pandemic and their preferred means to communicate with their orthodontists. Section 5 highlighted patients’ insights on postponing or discontinuing treatment, scheduled appointment intervals due to safety concerns, and their preferred choice of appliance during the closure. Section 6 focused on the clinic facilities, screening protocols, pretesting for COVID-19, PPE, and level of satisfaction on how treatment is handled in a restrictive environment. Finally, Section 7 contained questions from the Kessler Psychological Distress Scale (K10) [7, 8], which is a clinical measure of psychological symptoms. Specifically, this index is a validated 10-item scale, where each item has a 5-point scale that ranges from 1 (none of the time) to a score of 5 (all the time), with a total score range of 10–50. A total score of 10–19 was considered normal, while a score of 20–50 indicated anxiety and mental distress [28, 29]. The validity and reliability of this scale have been assessed in previous studies [19, 30].

Inclusion criteria were as follows: adult patients 18 years and older, undergoing active treatment with fixed orthodontic appliance therapy. Patients in retention or undergoing clear aligner therapy, with craniofacial syndromes and mental health disorders were excluded.

The following approach was used to recruit subjects for survey completion. Appointment schedules were screened daily in the duration between (late July and early October 2020) for eligible patients. An equal number of hard copy surveys to the eligible patients attending the clinic on each workday were prepared. The orthodontic provider (faculty or resident) was asked to distribute the anonymous paper survey to their eligible patients when they presented for their regular orthodontic appointment visits. The aims of the study were verbally explained to the eligible patient and a written explanation was provided as well. It was clearly stated that participation was voluntary. The estimate time for its completion (10 min) was pre-calculated using the surveylength.com instrument calculator, which takes into account the type, number of questions, and age of the targeted responders. Although the subjects had unlimited time to complete the survey, the average time to complete it matched what was pre-evaluated (10 min) as reported by the providers. A total of 200 surveys were distributed throughout the entire period, 149 were completed and returned onsite, and 5 were taken home and returned on a consecutive visit, the rest declined participation.

## Statistical analysis

Survey responses were summarized by frequencies and percentages or mean, standard deviation, and quartiles. The K10 questions were scored from the lowest (= 1) to the highest (= 5) degree of mental distress related to COVID-19 and the total was calculated, similarly for the Likert-scale questions to assess the level of confidence in resuming treatment. Cronbach’s alphas using all items and leaving one item out were reported for the internal reliability of K10 questions and questions on confidence in resuming treatment. Normal (≤ 19) and mental distress (> 19) groups based on the total K10 scores were compared for confidence in resuming treatment, assessed by individual questions and their combined total score, using two-sided Mann-Whitney U tests. P-values smaller than 5% were considered statistically significant. All the statistical analyses were performed in R version 3.6.1.

## Results

A total of 154 patients were surveyed between July and October 2020. The mean age of the participants was 29.30 (SD = 12.01) years. There were 95 females (62%) and 59 males (38%). Thirty nine percent (60/154) had been in treatment for 1 to 2 years and 42.9% (66/154) have not been seen by their orthodontist for at least 2 to 3 months (Table 1). Regarding the patients’ psychological aspects relevant to clinical orthodontics during the pandemic, the majority (61.69% or 95/157) did not report any pain or discomfort from the appliance during the pandemic, 48.7% (75/154) anticipated a delay in their treatment, while 85% (131/154) expected the delivery of excellent standard of care during treatment (Table 2).

**Table 1:** Section 1: Basic information

Question	Response	Number	% Frequency
**How long you have been in orthodontic treatment?**	> 3 years	33	21.4%
	2–3 years	23	14.9%
	1–2 years	60	39.0%
	1–12 months	37	24.0%
	0–1 month	1	0.6%
**How long you have been not seen by your orthodontist due to COVID-19?**	> 3 months	64	41.6%
	2–3 months	66	42.9%
	0–1 month	23	14.9%
	Unanswered	1	0.6%

**Table 2:** Section 2: Psychological aspects that are relevant to clinical orthodontics during the pandemic

Question	Response	Number	%
**I felt pain and discomfort from my appliance during the pandemic**	Worst possible	1	0.65%
	Severe pain	6	3.90%
	Moderate pain	22	14.29%
	Mild pain	25	16.23%
	No pain	95	61.69%
	Unanswered	5	3.25%
**How frequent was the pain/discomfort?**	Less than that	26	16.88%
	Everyday	8	5.2%
	Few times a week	16	10.39%
	Once a week	4	2.6%
	No discomfort or pain	95	59.1%
	Unanswered	2	1.3%
**I expect the standard of care delivered to be:**	Excellent	131	85%
	Good	21	14%
	Satisfactory	1	1%
	Unanswered	1	1%
**My orthodontic treatment will be delayed because of the pandemic**	Strongly agree	29	18.83%
	Agree	46	29.87%
	Disagree	28	18.18%
	Strongly disagree	8	5.19%
	Unanswered	43	27.92%
**How many extra months do you expect to stay in braces because of the pandemic?**	> 18 months	8	5.2%
	18 months	8	5.2%
	12 months	10	6.5%
	6 months	33	21.4%
	3 months	37	24.0%
	0	1	0.6%
	Unanswered	57	37.0%

A few respondents 21% (32/154) reported emergencies, which were considered of moderate intensity for the majority (11.04% or 17/32) and were mostly a “wire out or poking wire” (10.39% or 16/32) in nature. Emergencies were mainly handled by going to an emergency appointment (7.79 % or 12/32 in Table 3). For the patient/orthodontist follow-up section, 40.3% (62/154) preferred calling their orthodontists directly to report emergencies. The idea of tele-dentistry to monitor treatment seemed to be neutral for most (41.56% or 64/154). Approximately 73% (113/154) of the patients chose to be contacted by phone calls or text messages for following up with their treatment. The bulk 72.1% (111/154) favored to be checked upon monthly by their orthodontists and were willing to be seen monthly for regular checkups during the pandemic (73.4% or 113/154). When asked if they had a choice regarding the type of appliance (fixed vs. clear aligners) for their treatment during the pandemic, the majority 55% favored fixed appliances (Table 4).

**Table 3:** Section 3: Handling emergencies during the pandemic

Question	Response	Number	% Frequency
**Did you have any emergency during the pandemic?**	No	117	76%
	Yes	32	21%
	Unanswered	5	3%
**If you had any emergency, how do you classify your orthodontic emergency?**	Severe	7	4.55%
	Moderate	17	11.04%
	Minor	8	5.19%
	Unanswered	122	79.22%
**What was the nature of the emergency? (you can choose more than one)**	Loose appliance	1	0.6%
	Loose mini-screw	3	1.95%
	Detached braces	11	7.14%
	Wire out/poking	16	10.39%
	Unanswered	123	79.9%
**How did you handle your emergency?**	Called my provider and solved Issue over the phone	9	5.8%
	I handled the issue myself	11	7.1%
	Went for an emergency appointment	12	7.79%
	Unanswered	122	79.22%
**How urgent you felt the need to be seen by an orthodontist for your emergency during the pandemic?**	Extremely Necessarily	4	2.6%
	Necessary	17	11.0%
	Neutral	9	5.8%
	Unnecessary	2	1.3%
	Unanswered	122	79.2%
**I want to see my orthodontist for an emergency during the pandemic to:**	Handle the emergency	0	0%
	Handle the emergency and do the regular checkup appointment	0	0%
	Only for a consult, I am afraid to be operated on during the Pandemic	0	0%
	Unanswered	154	100%
**Do you believe your treatment would be delayed if the emergency were not handled by your orthodontist in the pandemic?**	Strongly agree	5	3.25%
	Agree	7	4.55%
	Neutral	12	7.79%
	Disagree	6	3.90%
	Unanswered	124	80.52%

**Table 4:** Section 4: Patient/orthodontist follow-up during the pandemic

Question	Response	Number	%
**What would your favorite mean to communicate with your orthodontist to inform him/her about your emergency during the pandemic?**	Social media groups	1	0.6%
	I Do Not Care	9	5.8%
	Call the operator at the hospital/beeper	19	12.3%
	Text message	54	35.1%
	Call my orthodontist directly	62	40.3%
	Unanswered	9	5.8%
**Do you like the idea of tele-dentistry to supervise the progress and integrity of the orthodontic appliance?**	Extremely useful	21	13.64%
	Useful	39	25.32%
	Neutral	64	41.56%
	Not useful	13	8.44%
	Extremely non-useful	8	5.19%
	Unanswered	9	5.84%
**Do you think that an emergency issue can be solved via tele-dentistry?**	Strongly agree	7	4.55%
	Agree	29	18.83%
	Neutral	70	45.45%
	Disagree	31	20.13%
	Strongly disagree	9	5.84%
	Unanswered	8	5.19%
**How would you like your orthodontist to follow up with your treatment during the pandemic?**	Tele-dentistry consults	5	3.25%
	Phone/text	13	8.44%
	Face to face	24	15.58%
	Text message	50	32.47%
	Phone	50	32.47%
	Other	8	5.19%
	Unanswered	4	2.60%
**How often would you like to connect with your orthodontist during the pandemic?**	Every 3 months	8	5.2%
	Every 2 months	23	14.9%
	Monthly	111	72.1%
	Unanswered	12	7.8%
**How often would you like to be seen for regular checkups during the pandemic?**	Every 12 weeks	3	1.9%
	Every 10 weeks	3	1.9%
	Every 8 weeks	9	5.8%
	Every 6 weeks	20	13.0%
	Monthly	113	73.4%
	Unanswered	6	3.9%
**If you had a choice of a type of an appliance to be used for your treatment during the pandemic would you go for:**	Aligners	44	29%
	Fixed braces	84	55%
	Unanswered	26	17%

Regarding the treatment protocols and the restrictive clinical environment during the COVID-19 pandemic, participants were extremely pleased with the restrictive protocols at the clinic 80.51% (124/154). Sixty-eight percent (105/154) agreed with the idea that telephone and pre-clinical appointment screenings are effective tools to prevent the spread of infection. Moreover, they recommended pre-testing the patients (81.81%), the staff, and orthodontists (85.06%) during the pandemic. Additionally, they preferred that their provider have a full PPE and a N95 mask on at the appointment (45.5%, Table 5).

**Table 5:** Section 5: Post-lockdown

Question	Response	Number	% Frequency
**I am extremely pleased with the restrictive protocols at the clinic because of the pandemic**	Strongly agree	71	46.10%
	Agree	53	34.42%
	Neutral	19	12.34%
	Disagree	5	3.25%
	Unanswered	6	3.90%
**I think that the telephone screening and the pre-clinical appointment screening is an effective tool to prevent the spread of infection**	Strongly agree	58	37.66%
	Agree	47	30.52%
	Neutral	33	21.43%
	Disagree	5	3.25%
	Strongly disagree	4	2.60%
	Unanswered	7	4.55%
**Pre-testing the patients for COVID 19 sign and symptoms (temperature) is very essential prior to the treatment during the pandemic**	Strongly agree	80	51.9%
	Agree	46	29.9%
	Neutral	17	11.0%
	Disagree	3	1.9%
	Strongly disagree	3	1.9%
	Unanswered	5	3.2%
**Pre-testing the staff and orthodontists is very essential during the pandemic**	Strongly agree	88	57.1%
	Agree	43	27.9%
	Neutral	12	7.8%
	Disagree	1	0.6%
	Strongly disagree	4	2.6%
	Unanswered	6	3.9%
**I prefer my orthodontist to:**	Wear full PPE without the N-95 mask	15	9.7%
	N-95 mask should be enough with eye cover	22	14.3%
	I personally do not care	34	22.1%
	Full PPE with the N-95 mask	70	45.5%
	Other	4	2.6%
	Unanswered	9	5.8%

The Cronbach alpha for the 10 items in the Kessler Psychological Distress Scale was 0.94 (95% CI: 0.93–0.95) considered excellent, with the mean total score of 13.16 (SD = 6.63, range: 5–50, Table 6). Similarly, the Cronbach alpha was acceptable (alpha = 0.65, 95% CI: 0.57–0.73) and the mean total score was 46.47 (SD = 4.75, range: 5–55) for the questions on confidence in resuming orthodontic treatments (Table 7). None of the individual items dropped significantly changed the Cronbach alpha for either scale. Overall, patients with mental distress had lower confidence in resuming orthodontic treatments than normal patients (P = 0.035, Table 8).

**Table 6:** Ranking of the 10 items in “Kessler’s Scale”

K10 all items	Mean	SD	25%	Median	75%	Complete observations
**Did you feel tired for no good reason?**	1.44	0.95	1	1	1	132 (86%)
**Did you feel nervous**	1.55	0.95	1	1	2	132 (86%)
**Did you feel so nervous that nothing could calm you down**	1.30	0.78	1	1	1	128 (83%)
**Did you feel that everything was an effort**	1.63	1.22	1	1	2	129 (84%)
**Did you feel sad that nothing could cheer you up**	1.24	0.77	1	1	1	132 (86%)
**Did you feel worthless?**	1.20	0.69	1	1	1	133 (86%)
**Did you feel restless or fidgety**	1.41	0.90	1	1	1	133 (86%)
**Did you feel so restless that you could not sit still**	1.23	0.64	1	1	1	132 (86%)
**Did you feel depressed?**	1.35	0.89	1	1	1	133 (86%)
**Did you feel hopeless?**	1.20	0.67	1	1	1	132 (86%)
**Total**	13.16	6.63	10	10	13	121 (79%)

**Table 7:** A descriptive summary for the 11 questions related to the confidence in resuming orthodontic treatment

Questions related to the level of confidence in resuming treatment in the pandemic	Mean	SD	25%	Median	75%	Complete observations
I am confident in resuming my orthodontic treatment and finishing it while at the pandemic	4.59	0.85	4	5	5	153 (99%)
I trust my orthodontist to deliver the same standard of care during the pandemic	4.61	0.81	4	5	5	153 (99%)
I have maintained the same standards of oral hygiene as instructed by my provider during the pandemic	4.54	0.80	4	5	5	153 (99%)
I have maintained the post-operative instructions and took care of my appliance during the pandemic	4.39	0.89	4	5	5	152 (99%)
It is necessary for an orthodontist to follow up on their patients during the pandemic or any similar emergencies	4.12	0.87	4	4	5	150 (97%)
Do you feel it is safe to see your orthodontist for full adjustment appointment during the pandemic?	4.21	0.82	4	4	5	150 (97%)
I like to postpone my treatment until the pandemic is over (negative correlated; coding reversed)	4.21	0.88	4	4	5	150 (97%)
I would like to discontinue my treatment and remove my braces because of the pandemic (negative correlated; coding reversed)	4.40	0.97	4	5	5	149 (97%)
I agree with the idea of reducing the number of visits to an orthodontic clinic during the pandemic (negative correlated; coding reversed)	2.95	1.11	2	3	4	149 (97%)
It is difficult to interact and communicate with my orthodontist while at the clinic during the pandemic (negative correlated; coding reversed)	3.98	0.89	4	4	4	133 (86%)
I am pleased with the way my appointment was handled during the pandemic.	4.39	0.87	4	5	5	146 (95%)
Total score	46.47	4.75	44	47	50	128 (83%)

**Table 8:** Comparison of orthodontic patients’ level of confidence in resuming treatment during the pandemic between normal and mental distress groups by the Kessler scale using two-sided Mann-Whitney U tests. *P* < 0.05*

Questions related to the level of confidence in resuming treatment in the pandemic	Normal: K10 total ≤ 19 (***n*** = 106)		Mental Distress: K10 total > 19 (***n*** = 15)		*P*-Value
	Mean ± SD	Median (25%, 75%)	Mean ± SD	Median (25%, 75%)	
**I am confident in resuming my orthodontic treatment and finishing it while at the pandemic**	4.61 ± 0.88	5.00 (5.00, 5.00)	4.44 ± 0.62	4.50 (4.00, 5.00)	0.038*
**I trust my orthodontist to deliver the same standard of care during the pandemic**	4.61 ± 0.83	5.00 (4.50, 5.00	4.56 ± 0.70	5.00 (4.00, 5.00)	0.468
**I have maintained the same standards of oral hygiene as instructed by my provider during the pandemic**	4.56 ± 0.79	5.00 (4.00, 5.00)	4.39 ± 0.92	5.00 (4.00, 5.00)	0.449
**I have maintained the post-operative instructions and took care of my appliance during the pandemic**	4.35 ± 0.93	5.00 (4.00, 5.00)	4.67 ± 0.49	5.00 (4.00, 5.00)	0.225
**It is necessary for an orthodontist to follow up on their patients during the pandemic or any similar emergencies**	4.08 ± 0.88	4.00 (4.00, 5.00)	4.44 ± 0.70	5.00 (4.00, 5.00)	0.086
**Do you feel it is safe to see your orthodontist for full adjustment appointment during the pandemic?**	4.26 ± 0.81	4.00 (4.00, 5.00)	3.89 ± 0.83	4.00 (3.25, 4.00)	0.056
**I like to postpone my treatment until the pandemic is over (negative correlated; coding reversed)**	4.27 ± 0.85	4.00 (4.00, 5.00)	3.83 ± 1.04	4.00 (3.00, 5.00)	0.071
**I would like to discontinue my treatment and remove my braces because of the pandemic (negative correlated; coding reversed)**	4.44 ± 0.95	5.00 (4.00, 5.00)	4.11 ± 1.13	5.00 (3.00, 5.00)	0.267
**I agree with the idea of reducing the number of visits to an orthodontic clinic during the pandemic (negative correlated; coding reversed)**	2.95 ± 1.08	3.00 (2.00, 4.00)	2.94 ± 1.30	3.00 (2.25, 4.00)	1.000
**It is difficult to interact and communicate with my orthodontist while at the clinic during the pandemic (negative correlated; coding reversed)**	4.07 ± 0.84	4.00 (4.00, 5.00)	3.41 ± 1.00	4.00 (2.00, 4.00)	0.004*
**I am pleased with the way my appointment was handled during the pandemic.**	4.43 ± 0.86	5.00 (4.00, 5.00)	4.11 ± 0.90	4.00 (4.00, 5.00)	0.081
**Total score**	46.78 ± 4.68	47.00 (45.00, 50.00)	44.41 ± 4.85	44.00 (42.00, 48.00)	0.035*

## Discussion

Adult orthodontic patients have peculiar psychological features as well as different treatment requirements compared to children and adolescents [31]. Methodological studies have shown that the data quality in surveys is influenced by the age of respondents [32]. Children were more likely to encounter problems with certain questions due to the lack of developed cognitive, communicative, and social skills which affects the different stages of the question-answer process [32]. Therefore, adults were chosen for the study cohort due to the reported inconsistency noticed in children’s answers to survey questions [13]. On the other hand, more females visited the clinic during the study period; therefore, we had a higher percentage of females among the respondents.

Our data showed that the lockdown forced 2 to 3 months of lapse in regular patients’ care. Since orthodontic therapy mainly consists of fixed appliances in the patient’s mouth, we expected a high percentage of emergencies during the lockdown. Surprisingly, we found that majority of our patients (61.69%) did not report any pain or inconvenience related to orthodontic treatment, and very few had emergencies (21%), which were mostly related to “poking wires and detached brackets.” Our survey was exclusive for patients treated with a fixed labial orthodontic appliance, which is the most common appliance in orthodontics [33], associated with orthodontic emergencies. Brackets or bondable buccal tubes loosening, and poking wires are the most common causes for orthodontic emergencies reported in the literature [25, 34]. In a survey study that evaluated the most common urgencies and emergencies in orthodontics during the COVID-19 pandemic, breakage of brackets, arch wires, tubes, and/or bands were the most common reasons for emergencies [24]. On the other hand, the minimal pain and emergencies reported might be attributed to the patients being adults instead of children or adolescents. The literature stated that reasons for the high treatment success in adults are increased cooperation, caring for the device, maintaining better oral hygiene, and compliance with the guidelines [35].

The survey revealed that patients would like to contact their orthodontists directly to report an emergency and follow up with their treatment. Their preferred method of contact was via text messages or phone calls. Success of treatment is highly impacted by the doctor-patient relationship [36]. Better communication is the key for improving the quality of care. The anxiety and psychological distress caused by the pandemic might be aggravated in the presence of health-related issues [14]. This might be allayed by effectual communication with the provider. Adherence to the orthodontic patients and keeping track on their treatment during the pandemic will positively influence treatment results by encouraging the patient to follow the instructions related to appliance wear and maintenance of oral hygiene [37]. Small gestures like regular check-up texts or calls can reduce the level of concern and give patients an uplift. Text messages and phone calls have been shown to be effective as a patient-chosen reminder method in orthodontics for appointments [38], oral hygiene instructions [39], and follow-up with treatment [40] and are the traditional common methods to communicate with patients. Thus, our patients picked text messages and phone calls and had a neutral attitude towards tele-orthodontics and remote monitoring, defined as the use of telecommunications to facilitate specific orthodontic information about the care to be delivered to the patients [17], which is novel and less commonly used. Despite the fact that tele-orthodontics is a viable tool to continue orthodontic care in the pandemic, and a future tool for orthodontic practice [26], our patients still preferred to come to their regular monthly checkups during the pandemic (73.4%).

Even though clear aligners have been proven to be more beneficial to the orthodontic patients during this unprecedented time [41], our patients favored to stay in fixed labial appliances. This may be attributed to satisfaction with their current progress of treatment with fixed appliances and possibly lack of awareness of the potential advantage of clear aligners to continue with the progress of treatment during a lockdown through remote delivery and monitoring by means of tele-dentistry [42].

The adherence to the most up-to-date strict clinical recommendations and protocols plays an integral role in orthodontic practice during the pandemic. Our patients were extremely content with the restrictive protocols and social distancing at the clinic which were in line with the Center for Disease Control and Prevention (CDC) and the American Dental Association (ADA) guidelines [43]. This indicates the high level of patients’ awareness of the severity of the disease and the importance of social distancing and personal protection. Public health and medical experts have clamored for increased coronavirus screening testing to control the COVID-19 pandemic [44]. Most of our patients were with the idea of pre-testing patients and the staff in the clinic and indicated that their orthodontist should have a full PPE and a N95 mask at the time of the appointment. The above-mentioned facts reflect the importance to stick to the new adaptations and changes due to the COVID-19 pandemic in the clinical practice of orthodontics. The World Health Organization (WHO) recommended health care workers involved in direct care should use gowns, gloves, medical mask, and eye protection (goggles or face shield). In aerosol-generating procedures, health care workers should use respirators (N95, FFP2, or equivalent standard), eye protection, gloves, and gowns (aprons should also be used if gowns are not fluid resistant) [45]. Some orthodontic procedures involve the generation of aerosols; therefore, it is essential to stick to the recommended PPE protocols to prevent the spread of the infection.

The Kessler Psychological Distress Scale is a nonspecific scale based on 10 questions about the level of anxiety and depressive symptoms a person may have experienced in the past 4 weeks. It has been extensively adopted to evaluate the anxiety of patients in the dental and medical fields [2, 15, 19]. Introduced in the annual US National Health Interview Survey [8], translated into eleven different languages [46, 47], and used as a screening tool for mental disorders in various situations [46, 48, 49] made it among the most reliable and valid tools to assess the psychological impact of the pandemic on orthodontic patients [19, 50]. Given its widespread utilization in survey research methodologies, we sought to integrate this tool in our survey design. Using the Kessler Scale (K10) , Xiong et al. [19] evaluated the mental health of orthodontic patients in China during the early phases of the pandemic and found that over one-third of the patients experienced mental distress. Earlier, Wu et al. [50] compared the psychological status between temporomandibular disorders (TMD), orthodontic patients, and the general population and concluded that TMD patients had a higher level of anxiety than the other two groups during the pandemic in China. Moreover, in another attempt to evaluate the impact of coronavirus pandemic on orthodontic appointments and orthodontic patients’ anxiety without the use of the Kessler Scale (K10), Cortin et al. [15] showed that patients willing to attend an orthodontic appointment had a lower level of anxiety than those who would not go. Therefore, we additionally aimed to assess the mental health of our orthodontic patients using the Kessler Psychological Distress Scale and correlate it to their level of confidence in resuming orthodontic treatment during and after the pandemic. The mean total score of the scale in our study was 13.15 (SD = 6.63, Table 6), which indicated a lack of anxiety and mental distress in our orthodontic patients. This contradicts with the recent findings of Xiong et al. [19] who found that over one-third of the orthodontic patients experienced mental distress during the early stage of the COVID-19 pandemic. The difference in the level of anxiety between both studies is attributed to the fact that both samples were interviewed at different points in time during the pandemic and in a different setup. Specifically, their survey was conducted online, majority of their sample answered the questions while at lockdown in the early stages of the pandemic when the level of anxiety from the pandemic was expected to be high and they were still reluctant to resume their treatment. In our sample, the questionnaires were handed in paper format to patients attending our clinic for regular checkup visits, which reflects a more optimistic outlook to the pandemic. Furthermore, patients visiting the orthodontic clinic for treatment are expected to have a lower level of anxiety and mental distress [19], and if we had the opportunity to interview patients who did not agree to see their orthodontist during the pandemic, we would likely have found higher scores for anxiety and distress.

On the other hand, 11 questions (items) that reflected the confidence of patients in resuming their orthodontic treatment while at the pandemic were picked from the survey, each item had a 5-point scale that ranged from 1 (low confidence in resuming treatment) to 5 (high confidence in resuming treatment). The mean total score for this scale was 46.46 (SD = 4.75) (Table 7), which indicated high confidence and positive attitude for the patients towards orthodontic treatment and the safety measures taken at the clinic during the pandemic. When comparing orthodontic patients’ level of confidence in resuming treatment during the pandemic between normal and mentally distressed groups by the Kessler scale (Table 8) a statistically significant relationship (P < 0.05) was observed for the association between higher anxiety and lower confidence in resuming treatment; but the anxiety level reported in our study was minimal despite the pandemic. Most of our patients still hold a positive attitude towards orthodontic treatment and are still willing to come and resume their treatment provided that the safety measures and restrictive protocols are implemented.

## Limitations

The convenience sampling for patients attending the same orthodontic clinic could have affected the generalizability of the findings. Normal and mental distress comparisons for orthodontic treatment confidence are underpowered. We were not able to anticipate the number of patients showing anxiety based on the K10 index, so therefore, the trend of lesser confidence in patients with higher anxiety should be further explored. Another limitation is that the survey questions measured outcomes experienced by patients who had relatively more positive outlook for the pandemic. Those patients considered orthodontic treatment essential during this unprecedented situation. The level of anxiety and confidence to resume orthodontic treatment might have been different if we interviewed those who have been hesitant to continue treatment after the lockdown, which constituted a small percentage in our clinic (we estimate less than 5% of our adult patients). Additionally, response rate for some questions in the section related to emergencies was low, since majority did not face any emergency that required immediate care. Given the limitations, our survey provides an insight into the awareness of most orthodontic patients about their treatment during the pandemic. Implementing measures to reinforce infection transmission control and increased social distancing are the new norm in the practice of orthodontics [18].

## Conclusions

This survey study sheds light on patients’ perceptions, preferences, and expectations in clinical orthodontics during the pandemic and provides guidelines for clinicians on the preferred protocols and practices for patients after clinics reopening. The direct communication with patients and regular checkup appointments were among their preferences. Keeping up with a strict clinical environment, social distancing, full PPE, pre-screening, and testing protocols are crucial points to be considered during the pandemic. The COVID-19 pandemic did not affect the mental health of orthodontic patients; many patients are still willing to be treated and expect a high level of standard of care to be delivered during the pandemic. A modest association between mental distress and reduced confidence in resuming treatment was found and needs to be further explored.

## Data Availability

The data underlying this article are available in the article and its online material.

## Abbreviations

ADA: American Dental Association
CDC: Center for Disease Control and Prevention
K10: Kessler Psychological Distress Scale
OSHA: Occupational Safety and Health Administration
PPE: Personal protection equipment
WHO: World Health Organization
